# Investigational Drug Available Directly from CDC for the Treatment of Infections with Free-Living Amebae

**Published:** 2013-08-23

**Authors:** Jennifer R. Cope

Infections caused by free-living amebae (FLA) are severe and life-threatening. These infections include primary amebic meningoencephalitis (PAM) caused by Naegleria fowleri* and granulomatous amebic encephalitis caused by *Balamuthia mandrillaris*† and *Acanthamoeba* species.§ Although several drugs have in vitro activity against FLA, mortality from these infections remains >90% despite treatment with combinations of drugs.

Miltefosine is a drug used to treat leishmaniasis and also has shown in vitro activity against FLA (1), but as an investigational drug, it has not been readily available in the United States. With CDC assistance, however, miltefosine has been administered since 2009 for FLA infections as single-patient emergency use with permission from the Food and Drug Administration. Although the number of *B. mandrillaris* and *Acanthamoeba* species infections treated with a miltefosine-containing regimen is small, it appears that a miltefosine-containing treatment regimen does offer a survival advantage for patients with these often fatal infections (2). Miltefosine has not been used successfully to treat a *Naegleria* infection, but the length of time it has taken to import miltefosine from abroad has made timely treatment of fulminant *Naegleria* infections difficult.

CDC now has an expanded access investigational new drug (IND) protocol in effect with the Food and Drug Administration to make miltefosine available directly from CDC for treatment of FLA in the United States. The expanded access IND use of miltefosine for treatment of FLA is partly supported by 26 case reports of FLA infection in which miltefosine was part of the treatment regimen (Division of Foodborne, Waterborne, and Environmental Diseases, National Center for Emerging and Zoonotic Infectious Diseases, CDC, unpublished data, 2013). Miltefosine generally is well-tolerated, with gastrointestinal symptoms the most commonly reported adverse effects. Clinicians who suspect they have a patient with FLA infection who could benefit from treatment with miltefosine should contact CDC to consult with an FLA expert. For diagnostic assistance, specimen collection guidance, specimen shipping instructions, and treatment recommendations, clinicians should contact the CDC Emergency Operations Center at 770-488-7100.

## Footnotes

* Additional information available at http://www.cdc.gov/parasites/naegleria.

† Additional information available at http://www.cdc.gov/parasites/balamuthia.

§ Additional information available at http://www.cdc.gov/parasites/acanthamoeba.
